# Differential effects of free viewing and goal-directed search on visual attention: An eye-tracking study

**DOI:** 10.1371/journal.pone.0357289

**Published:** 2026-09-15

**Authors:** Chenxiao Yue, Jiannan Chi, Jiahui Liu, Yixu Wang, Cong Zhang, Ming Cao

**Affiliations:** 1 School of Automation and Electrical Engineering, University of Science and Technology Beijing, Beijing, China; 2 Shunde Innovation School, University of Science and Technology Beijing, Foshan, China; University of Electronic Science and Technology of China, CHINA

## Abstract

In natural scenes, human gaze behavior is driven jointly by external stimuli from scene characteristics and individual internal goals. To investigate the influence of these two factors on gaze behavior, this paper systematically analyzes eye movement behavior under two task paradigms: free viewing and goal-directed search. First, the guiding effects of different hierarchical visual features on fixation behavior under the two task conditions are analyzed. Second, the differences in eye movement trajectory characteristics between the two task paradigms are compared. Finally, the CNN-TransGaze model is proposed to distinguish eye movement behavior patterns under different task conditions. The results show that shape, location and orientation features exhibit strong fixation attraction under both task paradigms, while semantic features are significantly enhanced in goal-directed search compared with free viewing. There are significant differences in eye movement behavior characteristics between the two task scenarios. Moreover, compared with traditional statistical feature extraction methods, deep learning models can capture latent high-dimensional features more effectively, thereby enabling accurate discrimination of distinct eye movement patterns. Efficient feature extraction from diverse eye movement patterns using deep learning enables the implementation of relevant applications, such as distinguishing healthy individuals from patients with abnormal eye movement patterns caused by neurodegenerative diseases.

## Introduction

When humans interact with their surroundings, the visual system need efficiently distribute limited cognitive resources across complex scenes. Attention, a core mechanism that prioritizes perceptual processing, is influenced by multiple factors such as perceptual features and task demands [1–3]. Understanding how these factors shape gaze allocation and visual exploration is therefore critical to uncovering human cognitive processes.

Visual processing is widely regarded as a hierarchical process [4], involving low-level perceptual feature extraction, mid-level structural analysis, and high-level semantic understanding [5]. Low-level perceptual features, such as color, luminance, and contrast, contribute to the formation of spatial priority representations that influence gaze allocation in visual scenes. Mid-level features involve edges, textures, and shape structures, which help describe the spatial organization of scenes and object contours. At a higher level, semantic information, such as object categories and scene semantics, also play an important role in guiding visual selection. Many theories of visual attention attempt to explain how these visual features interact to guide gaze behavior. For example, Feature Integration Theory [6] proposes that basic visual features are processed automatically in a pre-attentive stage, after which attention integrates these features into coherent perceptual objects. Furthermore, computational saliency models suggest that multiple visual features can be integrated to generate spatial priority maps that estimate regions more likely to attract gaze in natural scenes [7,8].

In addition to visual features, contextual factors such as viewing tasks also influence how observers allocate attention to different visual information [9]. Visual attention is commonly considered to arise from the interaction between bottom-up and top-down mechanisms. Bottom-up processes are primarily stimulus-driven, associated with feature contrast and saliency in the visual input, while top-down processes are influenced by task goals, prior knowledge, and observer expectations [10]. The relative emphasis of these two mechanisms varies across different task contexts, leading to differences in the extent to which different features guide attention.

Most existing studies on visual saliency focus on the free-viewing paradigm and primarily model gaze guidance driven by bottom-up visual features, with limited consideration of top-down task modulation during goal-directed search. While prior evidence has shown that low-level features alone cannot fully explain human fixation patterns, a systematic and quantitative comparison of gaze-guiding strength across hierarchical visual features under different task conditions remains lacking. In parallel, conventional visual search experiments typically rely on simplified stimuli composed of one or a few isolated features and evaluate performance using reaction time. Such designs deviate from real-world visual environments, where low-, mid-, and high-level features coexist and interact. As a result, there is still no unified framework that quantitatively characterizes how different feature hierarchies compete to guide human gaze in complex natural scenes. Furthermore, most existing analyses rely on basic statistical descriptors, with limited exploration of data-driven approaches for extracting task-specific temporal representations from scanpaths.

Against this backdrop, this study formulates three progressive research questions. First, when multi-level visual features coexist in natural scenes, which feature hierarchy exerts the strongest gaze-guiding effect, and how does this effect differ between free viewing and goal-directed search? Second, do these two task paradigms give rise to fundamentally distinct gaze patterns at the behavioral level? Third, if such differences exist, what core eye-movement representations distinguish the two patterns, and can temporal deep learning models more effectively capture these latent spatiotemporal dynamics compared with traditional approaches based on handcrafted features?

**Fig 1:**
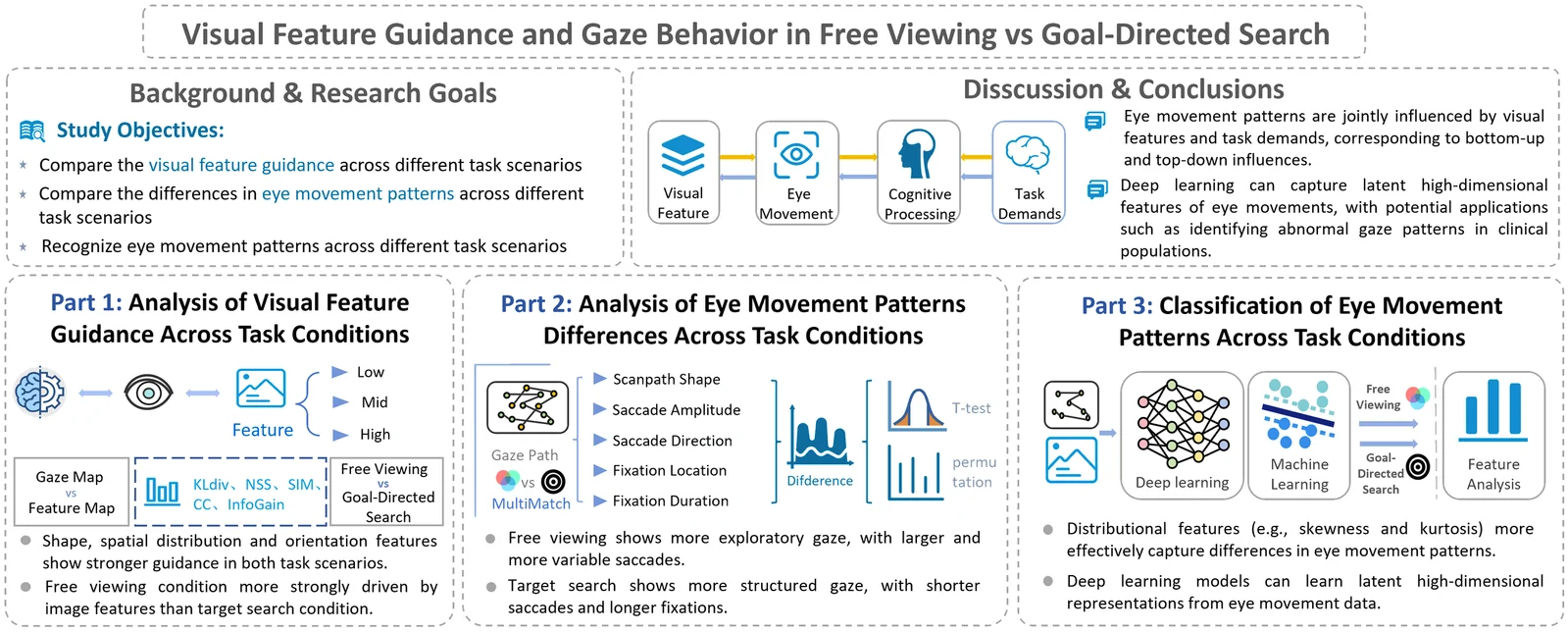
Graphical abstract. This graphical abstract presents the full research framework, including study goals, three analytical parts, key results, and overall conclusions on how visual features and task demands shape gaze in free viewing and goal-directed search. All analysis code is available at https://github.com/jianxueshen/TP-vs-BU-attention-gaze-code, and the supporting dataset is available at https://zenodo.org/records/17060884.

To address these questions, we propose an integrated analytical framework spanning three complementary levels: feature-level mechanisms, behavioral-level quantification, and model-level temporal representation learning. At the feature level, we introduce standardized metrics to quantify the spatial consistency between feature-specific saliency maps and empirical fixation distributions, enabling direct comparison of gaze-guiding strength across feature hierarchies and task conditions. At the behavioral level, we characterize scanpath differences using multi-dimensional eye-movement metrics and statistical analyses to reveal systematic task-dependent variations. At the model level, we construct classification models to investigate whether traditional machine learning and temporal deep learning architectures can effectively extract and discriminate task-specific eye-movement patterns, thereby uncovering latent spatiotemporal representations beyond conventional statistical features. The overall structure of the article is shown in Fig 1.

## Methods

The methodological part of this study consists of three components. First, saliency maps are generated for individual visual features, and their distributional differences from human fixation heatmaps are computed to quantify the correspondence between visual features and fixation locations and evaluate their guiding effects on fixation clustering. Second, eye movement trajectories under two task conditions, free viewing and goal-directed search, are compared to examine how different task contexts influence visual behavior patterns. Third, a neural network model is constructed to recognize and classify eye movement trajectories across different task contexts.

This study uses the publicly available COCO-Search18 dataset for analysis [11]. The dataset contains eye movement data collected under two task conditions (free viewing and goal-directed search). A total of 10 participants searched for 18 target categories across 6,202 natural scene images while their gaze behavior was recorded using a high-precision eye tracker with a sampling rate of 1 kHz. The experiment lasted approximately 12 hours in total and yielded about 300,000 valid search fixations.

### Consistency analysis between different visual feature distributions and fixation distributions

**Fig 2:**
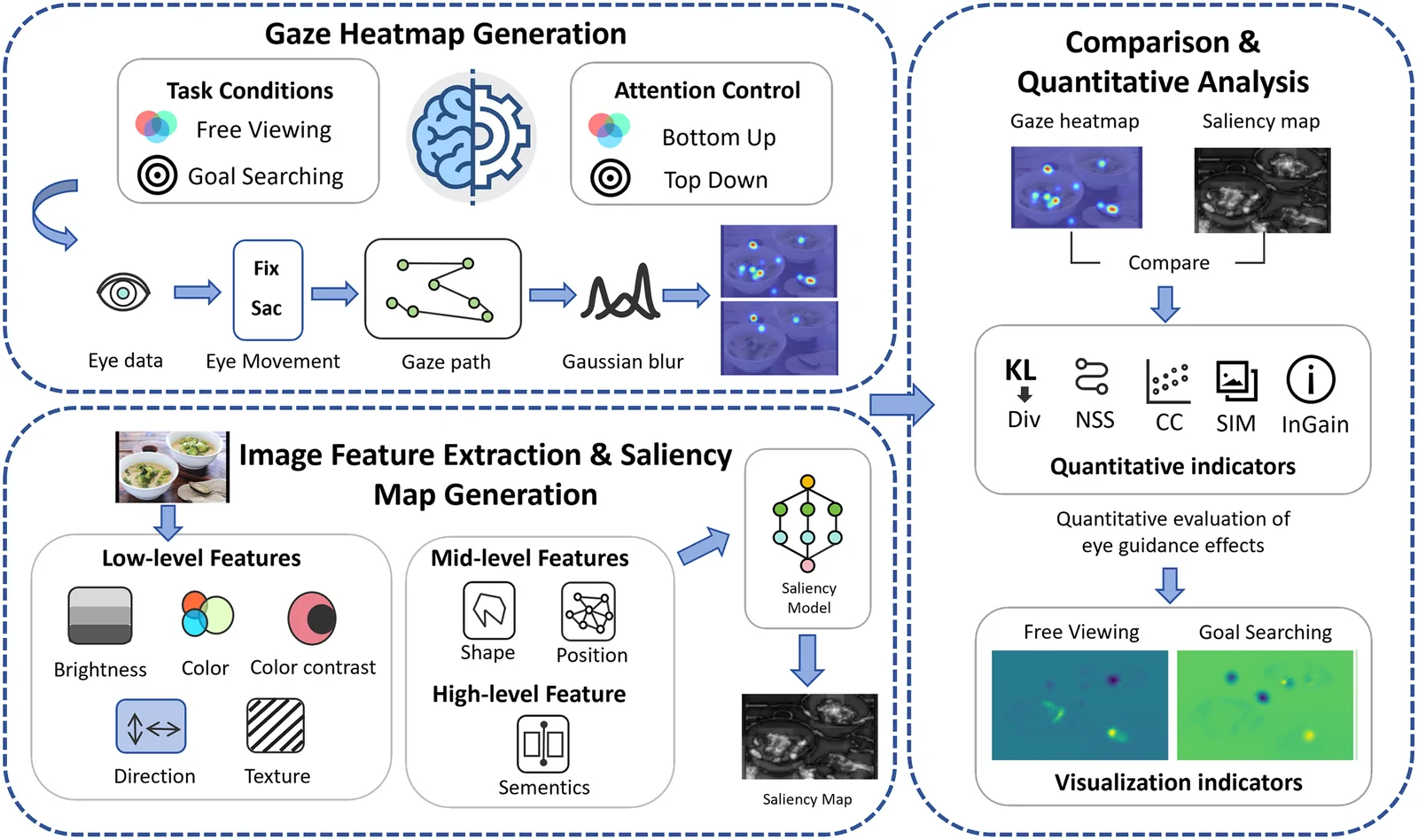
Analytical pipeline for consistency analysis between visual feature distributions and fixation distributions. This figure illustrates the complete processing pipeline for quantifying how visual features guide human gaze, covering gaze heatmap construction, multi-level image feature extraction, saliency map generation, and cross-task quantitative comparison.

To analyze the consistency between feature distributions and fixation distributions and to further evaluate the guiding effects of different visual features on gaze allocation, fixation detection algorithms are first applied to segment fixations and saccades from the raw eye-tracking data, and gaze distribution heatmaps are generated using Gaussian smoothing and pseudo-color visualization. Second, low-level, mid-level, and high-level visual features are extracted from the original images and transformed into corresponding feature-specific saliency maps using saliency map generation models. Finally, the fixation heatmaps are compared with the saliency maps generated from each visual feature, and multiple evaluation metrics are employed to quantify the correspondence between visual features and gaze distributions. The complete analytical pipeline of the proposed method is illustrated in Fig 2.

This study draws on Feature Integration Theory [12] and research on hierarchical visual processing [5] to extract multi-level visual features from images. These features are categorized into low-level, mid-level, and high-level visual features.

**Low-level features** include luminance, color, color contrast, orientation, and texture:

- **Luminance features:** Image brightness is represented using grayscale values. Based on histogram analysis, pixels are categorized into low-, medium-, and high-luminance regions.
- **Color features:** Pixel values are extracted from the RGB channels to describe the distribution of different color components in the image.
- **Color contrast features:** The image is first converted into the HSV color space, and K-means clustering (k = 7) is applied to the Hue channel to segment hue regions. The chromatic differences are then weighted according to the spatial proportions of each region to generate a color contrast saliency map.
- **Orientation features:** The Sobel operator is used to compute the horizontal and vertical gradients of the image in order to extract the primary edge orientation information. The gradient map is then normalized to generate an orientation saliency map.
- **Texture features:** Texture information is extracted using the Local Binary Pattern (LBP) method, which describes local grayscale pattern distributions and characterizes the structural complexity of the image [13].

**Mid-level features** mainly describe shapes and spatial structures in the image:

- **Shape features:** The Canny edge detection algorithm is used to extract edge contours from images, thereby characterizing the geometric contour features of objects in the image.
- **Spatial position features:** Spatial position features are extracted using the ORB (Oriented FAST and Rotated BRIEF) algorithm, which obtains the scale, orientation, response strength, octave level and descriptor information of keypoints in the image. By traversing and plotting the positional and directional relationships between each keypoint, a spatial position map is constructed to characterize the spatial distribution features of the image.

**High-level features** focus on the semantic information of images:

- **Semantic Features:** A pretrained Detectron2 model is used to perform object detection and semantic segmentation on the image, identifying different semantic object categories. Object masks obtained from segmentation are then used to generate semantic feature maps, representing the spatial distribution of semantic objects within the image.

**Fig 3:**
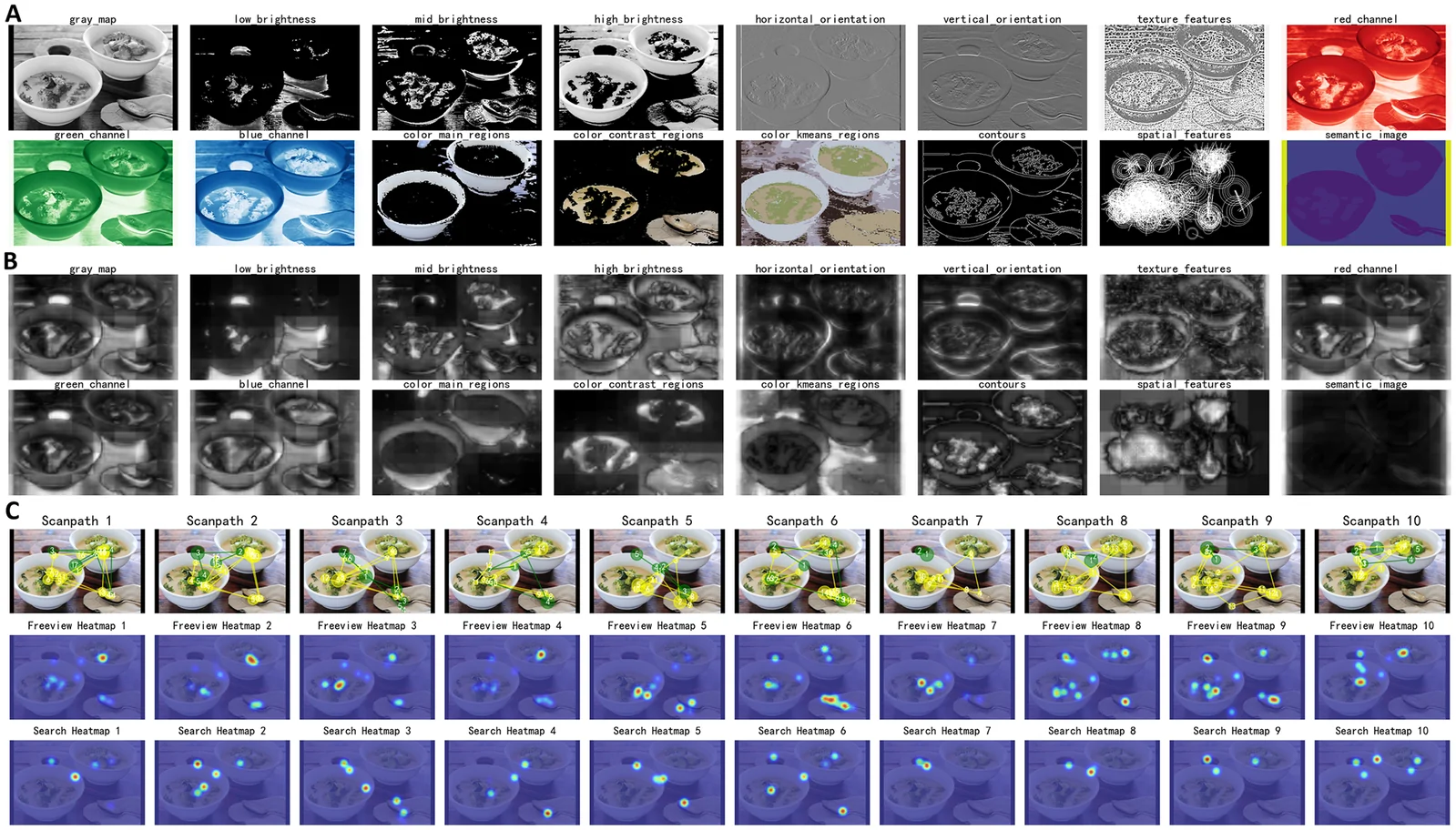
Examples of extracted feature maps, corresponding feature-based saliency maps, and gaze trajectories with fixation heatmaps. (A) Extracted image feature maps. (B) Corresponding feature-based saliency maps. (C) Eye-movement trajectories and fixation heatmaps.

All extracted feature maps are standardized and normalized before being used for saliency map generation and comparison with fixation heatmaps. Fig 3A presents representative feature maps extracted from a sample image, ordered from the top-left to the bottom-right: grayscale; luminance maps (low, medium, high); horizontal orientation map; vertical orientation map; texture map; RGB channel maps; dominant color map; color-contrast map; color-region segmentation map; shape map; spatial-position map; and semantic map.

Due to substantial differences among various feature maps, directly comparing them with gaze-derived heatmaps is not readily comparable. In this study, all extracted feature maps were processed using z-score normalization and feature-specific saliency maps were generated using the Itti model [14]. The results are shown in Fig 3B, with the top-left to bottom-right order corresponding to that in Fig 3A.

This study generated fixation heatmaps based on gaze data provided by COCO-Search. First, fixation point maps were created in image coordinates according to fixation positions and durations. To better approximate the spatial perception characteristics of human vision, the fixation maps were smoothed using a two-dimensional Gaussian filter. The resulting grayscale maps were then converted to pseudo-color images (JET colormap) to differentiate fixation intensity regions: high-intensity areas are represented in warm colors, and low-intensity areas in cool colors. As shown in Fig 3C, the figure presents the eye trajectories and generated fixation heatmaps for 10 participants on one example image from the dataset. The first row shows the eye trajectories overlaid on the image, with yellow representing free viewing and green representing goal-directed search. The second row displays the fixation heatmap for free viewing, and the third row shows the fixation heatmap for goal-directed search.

To analyze the relationship between feature-based saliency maps and human gaze distributions, the generated feature saliency maps were compared with the corresponding human fixation heatmaps. Five commonly used saliency evaluation metrics were employed for quantitative assessment [8]. The specific metrics are described as follows:

- **Kullback–Leibler Divergence (KLdiv):** KLdiv measures the difference between the global probability distributions of saliency map and fixation heatmap. A smaller KLdiv value indicates a higher similarity between the two distributions across the entire image [15].
- **Normalized Scanpath Saliency (NSS):** NSS assesses the feature saliency response at human fixation locations. Higher NSS values indicate that human fixations are more likely to fall on regions with strong feature saliency [16].
- **Pearson Correlation Coefficient (CC):** CC measures the linear correlation between feature saliency maps and fixation heatmaps at the pixel level. Values closer to 1 indicate higher consistency in their spatial distributions [15].
- **Similarity (SIM):** SIM calculates the spatial overlap between saliency maps and fixation heatmaps using histogram intersection. Higher SIM values indicate greater spatial coincidence between salient regions and fixation hotspots [7].
- **Information Gain (IG)** [17]: IG is used to evaluate the guidance of image features on fixation distributions while accounting for center bias in human gaze behavior. Since human fixations tend to concentrate near the center of an image, a Gaussian distribution centered on the image is used as the center-bias baseline model. IG compares the consistency between the feature-based saliency distribution and the actual fixation distribution with that between the center-bias model and the fixation distribution, thereby measuring the extent to which image features guide human fixations beyond center bias.

Mathematical Definitions:


$$\begin{array}{c} \text{KL}\left(F,G\right)=\sum_{i}G_{i}log\left(\frac{\varepsilon +G_{i}}{\varepsilon +F_{i}}\right) \end{array}$$ (1)

$$\begin{array}{c} \begin{array}{c} NSS\left(F,G\right)=\frac{\text{1}}{N}\sum_{i}F_{i}G_{i}\\ where,N=\sum_{i}G_{i},and,F=\frac{F-\mu_{F}}{\delta_{F}} \end{array} \end{array}$$ (2)

$$\begin{array}{c} CC\left(F,G\right)=\frac{\delta \left(F,G\right)}{\delta \left(F\right)\times \delta \left(G\right)} \end{array}$$ (3)

$$\begin{array}{c} \begin{array}{c} SIM\left(F,G\right)=\sum_{i}min\left(F_{i},G_{i}\right)\\ where,\sum_{i}F_{i}=\sum_{i}G_{i}=1 \end{array} \end{array}$$ (4)

$$\begin{array}{c} IG\left(F,G\right)=\frac{\text{1}}{N}\sum_{i}G_{i}\left[{log}_{\text{2}}\left(\varepsilon +F_{i}\right)-{log}_{\text{2}}\left(\varepsilon +B_{i}\right)\right] \end{array}$$ (5)

Here, F represents the feature saliency map, G represents the fixation heatmap derived from human eye-tracking data, and B indicates the prior center bias (modeled as a Gaussian distribution centered on the image).

### Eye movement trajectory comparison method

To systematically compare eye movement trajectories between free viewing and goal-directed search tasks, this study used the MultiMatch method [18,19] to analyze the two task conditions. We calculated five key metrics: scanpath shape, saccade amplitude, saccade direction, fixation position, and fixation duration.

- **Scanpath shape** describes the overall structure of a gaze trajectory and reflects the general pattern of visual exploration.
- **Saccade amplitude** represents the Euclidean distance between consecutive fixations, showing how widely visual exploration is spread across the scene.
- **Saccade direction** describes the direction of gaze shifts, revealing spatial distribution tendencies of visual attention.
- **Fixation position** is computed as the Euclidean distance between matched fixation points, showing differences in where spatial attention falls across the image.
- **Fixation duration** measures the dwell time at each fixation, indicating which regions receive more focused visual attention.

Although saccade amplitude and fixation position are computed similarly, they reflect different visual properties: saccade amplitude highlights the scope of visual exploration, while fixation position focuses on differences in attended spatial areas.

### Neural network classification approach

To examine whether gaze trajectories contain discriminative information that can differentiate between viewing tasks, we employed multiple machine learning algorithms to classify eye movement data, including Support Vector Machines (SVM), Decision Trees (DT), Random Forests (RF), Multilayer Perceptron (MLP), Logistic Regression (LR), Naive Bayes (NB), k-Nearest Neighbors (k-NN), and Adaptive Boosting (AdaBoost). Parameter settings are shown in Table 1.

**Table 1:** Parameter settings for each classification model.

Model	Key Parameters
SVM	kernel=rbf, C=10, gamma=scale
Decision Trees	max_depth=5, random_state=42
Random Forests	n_estimators=100,max_depth=5, random_state=42
MLP	hidden_layer_sizes=(50), max_iter=200
AdaBoost	n_estimators=100, random_state=42
Regression	solver=lbfgs,max_iter=200, random_state=42
kNN	n_neighbors=5, metric=minkowski, p=2

For the machine learning methods, the input features consisted of eight statistical eye movement descriptors, including the mean, variance, skewness, and kurtosis of fixation duration and saccade amplitude.

**Fig 4:**
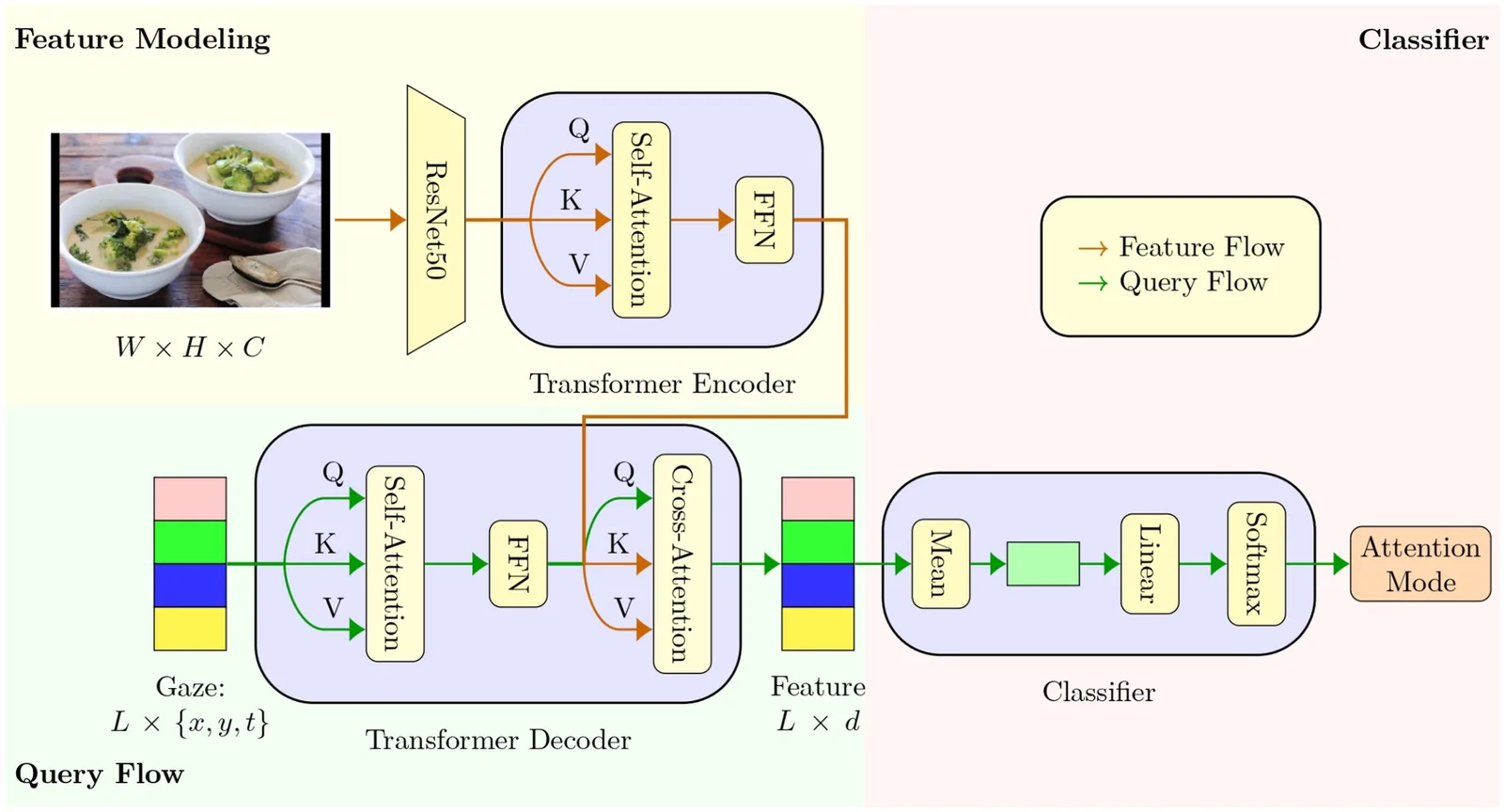
Overview of the proposed CNN–transformer framework for classifying eye movement trajectories across viewing tasks. This schematic depicts our end-to-end CNN–Transformer classifier: ResNet50-encoder extracts image features, Transformer decoder models gaze sequences and fuses cross-modal signals via cross-attention, and the classifier predicts viewing task types. Orange arrows denote visual feature flow, green arrows denote gaze query flow.

In addition, we developed a neural network model based on CNN and Transformer architectures to model temporal patterns in eye movement sequences and classify eye movement trajectories under different viewing task conditions. As shown in Fig 4, the model consists of an image encoder and a sequence decoder. The encoder first extracts visual contextual features using a ResNet-50 backbone, followed by a Transformer encoder to further model visual representations. The decoder then models temporal dependencies between fixation points using multi-head self-attention mechanisms. The final classification is achieved by integrating visual features with eye movement features.

The network includes 3 encoder layers and 3 decoder layers, each employing 8 attention heads with a hidden dimension of 512. The maximum eye movement sequence length is set to 7 fixation events. The model is trained using the AdamW optimizer with a cross-entropy loss function.

## Results

### Results of feature guidance analysis

**Fig 5:**
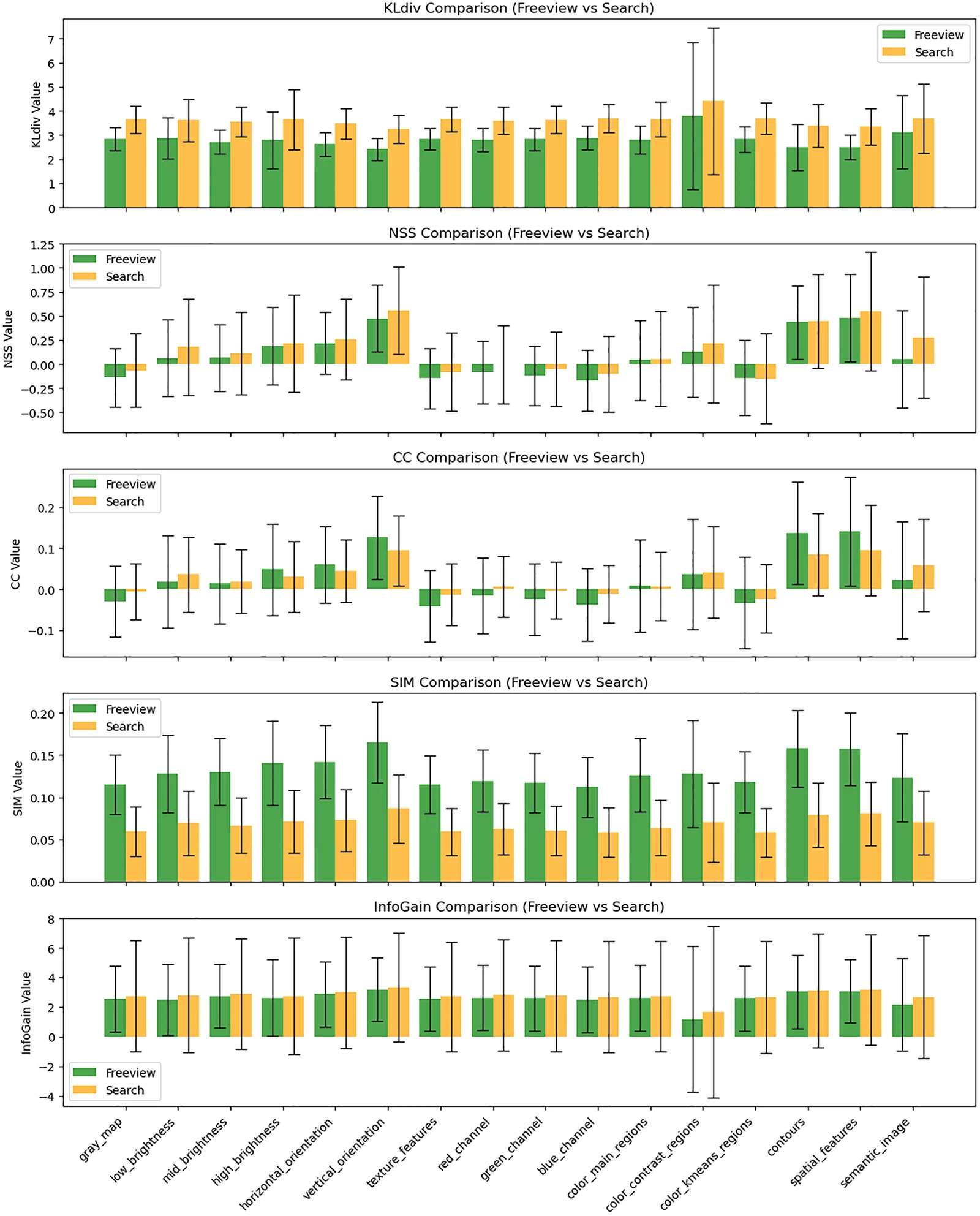
Five-metric comparison across image features. bar plots comparing KL divergence, NSS, CC, SIM, and InfoGain between free viewing (green bars) and goal-directed search (orange bars) conditions for low-, mid-, and high-level visual features. Error bars denote the standard deviation of metric values across all stimulus images.

Fig 5 visualizes the same quantitative data summarized in Table 2, presenting five distribution consistency metrics calculated between saliency maps of all 16 extracted visual features and human recorded fixation heatmaps, under free viewing (green bars) and goal-directed search (yellow bars) experimental conditions. For metric interpretation: smaller KL divergence (KLdiv) values, as well as larger NSS, CC and SIM values, indicate stronger distribution alignment between visual features and human fixations; higher Information Gain (InfoGain, IG) values represent better consistency after eliminating the central bias inherent to human gaze. The results indicate that KL divergence and SIM show similar distribution patterns across features, and feature distributions under the free-viewing condition are more consistent with fixation distributions. NSS and CC also display comparable trends across features, with shape–spatial position and orientation-related features exhibiting relatively stronger positive correlations with fixation distributions.

**Table 2:** Comparison of feature responses between free viewing (FV) and goal-directed search (SE) across different metrics (Mean ± Std).

Feature	KLdiv		CC		SIM		NSS		InfoGain	
	FV	SE	FV	SE	FV	SE	FV	SE	FV	SE
Gray	2.85 ± 0.47	3.66 ± 0.57	−0.03 ± 0.09	0.01 ± 0.07	0.12 ± 0.04	0.06 ± 0.03	−0.14 ± 0.30	−0.06 ± 0.38	2.56 ± 2.20	2.74 ± 3.74
LowBright	2.89 ± 0.85	3.62 ± 0.88	0.02 ± 0.11	0.04 ± 0.09	0.13 ± 0.05	0.07 ± 0.04	0.06 ± 0.40	0.18 ± 0.50	2.50 ± 2.37	2.79 ± 3.86
MidBright	**2.72 ± 0.49**	**3.56 ± 0.61**	0.01 ± 0.10	0.02 ± 0.08	0.13 ± 0.04	0.07 ± 0.03	0.07 ± 0.35	0.11 ± 0.43	**2.75 ± 2.16**	**2.88 ± 3.72**
HighBright	2.80 ± 1.18	3.65 ± 1.26	**0.05 ± 0.11**	0.03 ± 0.09	**0.14 ± 0.05**	**0.07 ± 0.04**	**0.19 ± 0.40**	0.21 ± 0.50	2.63 ± 2.58	2.74 ± 3.93
HorizOrient	**2.62 ± 0.51**	**3.49 ± 0.63**	**0.06 ± 0.09**	**0.04 ± 0.08**	**0.14 ± 0.04**	**0.07 ± 0.04**	**0.22 ± 0.32**	**0.26 ± 0.42**	**2.89 ± 2.20**	**2.98 ± 3.76**
VertOrient	**2.42 ± 0.47**	**3.25 ± 0.57**	**0.13 ± 0.10**	**0.09 ± 0.09**	**0.17 ± 0.05**	**0.09 ± 0.04**	**0.48 ± 0.34**	**0.56 ± 0.45**	**3.18 ± 2.15**	**3.33 ± 3.68**
Texture	2.85 ± 0.44	3.67 ± 0.52	−0.04 ± 0.09	−0.01 ± 0.08	0.12 ± 0.03	0.06 ± 0.03	−0.15 ± 0.32	−0.08 ± 0.40	2.56 ± 2.18	2.72 ± 3.69
Red	2.81 ± 0.48	3.61 ± 0.57	−0.02 ± 0.09	0.01 ± 0.07	0.12 ± 0.04	0.06 ± 0.03	−0.08 ± 0.33	−0.00 ± 0.41	2.62 ± 2.19	2.81 ± 3.73
Green	2.83 ± 0.47	3.65 ± 0.57	−0.02 ± 0.09	−0.00 ± 0.07	0.12 ± 0.04	0.06 ± 0.03	−0.12 ± 0.31	−0.05 ± 0.39	2.59 ± 2.20	2.75 ± 3.74
Blue	2.89 ± 0.49	3.70 ± 0.58	−0.04 ± 0.09	−0.01 ± 0.07	0.11 ± 0.04	0.06 ± 0.03	−0.17 ± 0.32	−0.10 ± 0.39	2.50 ± 2.23	2.68 ± 3.76
MainColor	2.81 ± 0.59	3.66 ± 0.70	0.01 ± 0.11	0.01 ± 0.08	0.13 ± 0.04	0.06 ± 0.03	0.04 ± 0.41	0.06 ± 0.49	2.62 ± 2.22	2.73 ± 3.74
ContrastColor	3.81 ± 3.05	4.40 ± 3.05	0.04 ± 0.13	0.04 ± 0.11	0.13 ± 0.06	0.07 ± 0.05	0.13 ± 0.47	0.21 ± 0.61	1.18 ± 4.92	1.66 ± 5.78
KMeansColor	2.83 ± 0.52	3.71 ± 0.65	−0.03 ± 0.11	−0.02 ± 0.08	0.12 ± 0.04	0.06 ± 0.03	−0.14 ± 0.39	−0.15 ± 0.47	2.59 ± 2.21	2.67 ± 3.76
Contours	**2.51 ± 0.95**	**3.39 ± 0.90**	**0.14 ± 0.12**	**0.08 ± 0.10**	**0.16 ± 0.05**	**0.08 ± 0.04**	**0.43 ± 0.38**	**0.44 ± 0.49**	**3.05 ± 2.48**	**3.12 ± 3.84**
Spatial	**2.49 ± 0.51**	**3.36 ± 0.76**	**0.14 ± 0.13**	**0.09 ± 0.11**	**0.16 ± 0.04**	**0.08 ± 0.04**	**0.48 ± 0.45**	**0.55 ± 0.62**	**3.08 ± 2.16**	**3.17 ± 3.74**
Semantic	3.12 ± 1.52	3.70 ± 1.44	0.02 ± 0.14	**0.06 ± 0.11**	0.12 ± 0.05	0.07 ± 0.04	0.05 ± 0.51	**0.28 ± 0.63**	2.16 ± 3.12	2.68 ± 4.13

**Note:** The top five features in each column that exhibit the strongest visual guidance are highlighted in bold. FV stands for free viewing, and SE denotes goal-directed search.

### Kullback-Leibler divergence analysis

KLD was used to quantify the difference between the global probability distributions of feature saliency maps and fixation heatmaps. The statistical results in Table 2 show that, across all feature types, KLD values under the target-search task are generally higher than those under the free-viewing task.

Across different feature categories, mid-level features (such as contour and spatial features) exhibit relatively lower KLD values. Low-level features (including orientation, brightness, and color channels) show moderate KLD values, whereas color contrast regions within the low-level features and the high-level semantic feature present relatively higher KLD values. In addition, these features also show relatively large standard deviations.

**Fig 6:**
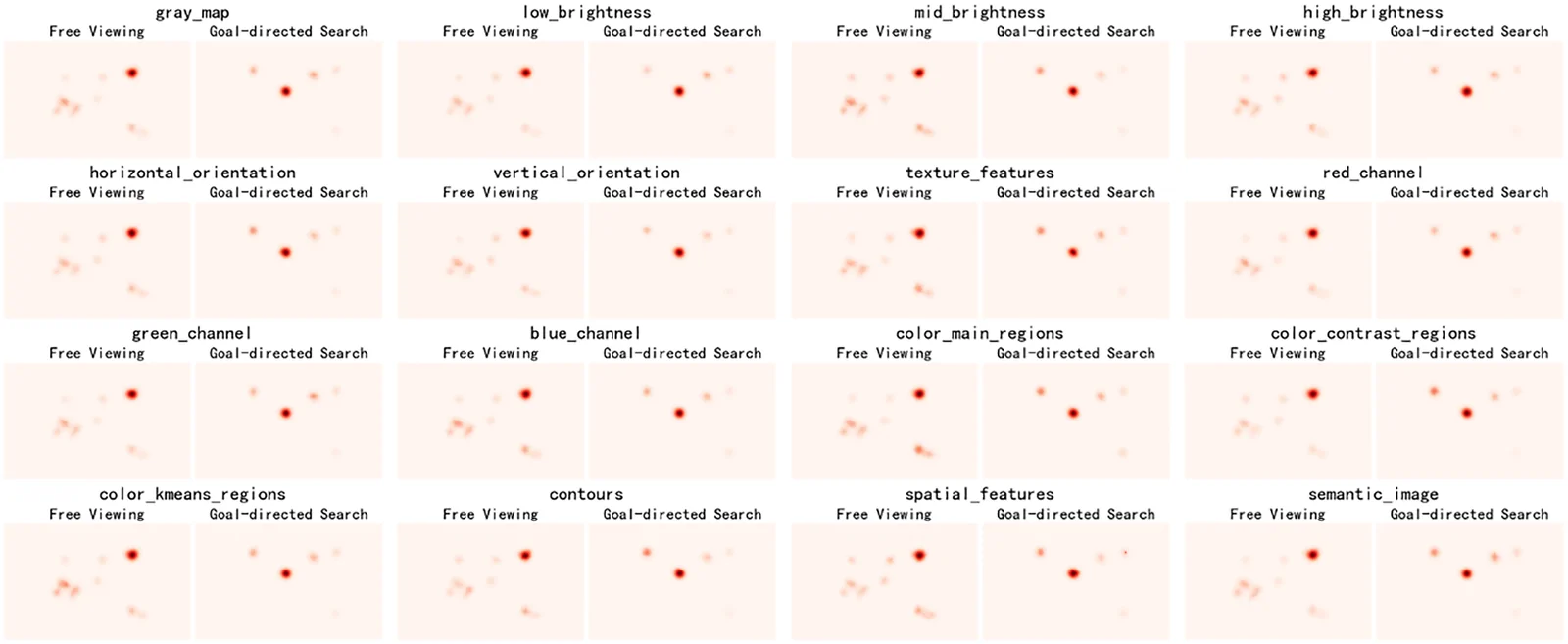
Visualized KL divergence maps of saliency and fixation distributions for free viewing vs goal-directed search. Pixel-wise KL divergence heatmaps measuring distribution discrepancy between multi-level visual feature saliency maps and human fixation heatmaps, paired side-by-side for free viewing (left) and goal-directed search (right) conditions on one representative stimulus from a single participant.

Fig 6 presents an example of the spatial distribution of KL divergence for different features under the free-viewing and target-search tasks, computed using the fixation heatmap of a single participant on an example image (the original image shown in Fig 3). This example image was randomly selected from the full COCO-Search18 dataset without subjective manual filtering to eliminate selection bias. The color scale uses the Reds colormap, where darker colors indicate higher KL divergence values, highlighting spatial regions with large dispersion between visual feature distribution and human fixation distribution. As shown in Fig 6, the spatial distributions of KL divergence are generally similar across different features, while under the target-search task the spatial distribution of high KL divergence regions appears relatively less extensive.

### Correlation coefficient analysis

CC quantifies the pixel-wise linear correspondence between feature maps and fixation heatmaps. A larger positive CC denotes stronger fixation attraction, while a larger absolute negative CC indicates more prominent inhibition.

As shown in Table 2, except for low-medium brightness and semantic features, all features have greater absolute CC values under free viewing than target-search tasks, indicating stronger visual feature influence in free viewing. Monochromatic R/G/B channels, texture, and color segmentation features show weak negative correlations with fixation distribution, indicating inhibitory gaze guidance effects. All visual cues above are global feature maps algorithmically extracted following the pipelines described in Section 2.1, without manual constraints on specific geometric shapes, spatial layouts or semantic object categories. Mid-level shape features (Canny edge contours) and spatial position features (ORB keypoint-based spatial maps) respectively exhibit strong positive correlations under both viewing tasks. High-level semantic features generated from Detectron2 semantic segmentation also deliver more prominent positive correlations during goal-directed search, a pattern driven by the essential function of object recognition for target localization.

**Fig 7:**
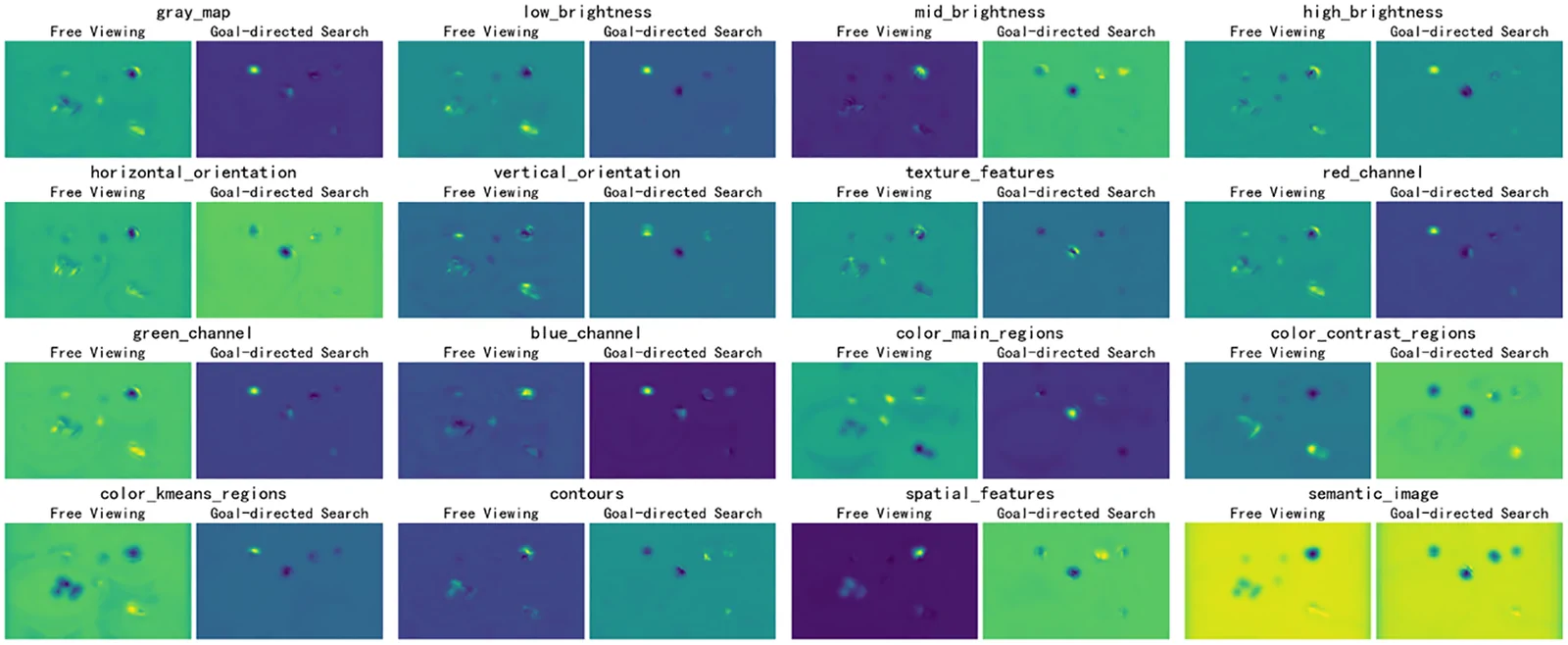
Visualized CC correlation maps of saliency and fixation distributions for free viewing vs goal-directed search. Spatial correlation heatmaps derived from the CC metric to quantify pixel-level similarity between multi-scale feature saliency maps and eye fixation heatmaps. Each feature type contains two subplots contrasting free viewing and goal-directed search gaze patterns from one sample subject and stimulus image.

Fig 7 illustrates an example of the spatial distribution of CC values for different features under the free-viewing and target-search tasks, computed from the fixation heatmap of a single participant on an exemplary image. All visualization results share the identical sample stimulus shown in Fig 3. The image uses the viridis color map: bright yellow indicates stronger positive correlation (i.e., higher CC values), meaning the spatial distribution of visual features and human fixations are strongly positively correlated, while dark purple indicates negative correlation (i.e., lower CC values), reflecting weak matching between feature and fixation distributions. For shape and spatial position features, in most regions under the target-search condition, the CC values are significantly higher than those under the free-viewing condition, which may be related to the role of shape and spatial position in target recognition.

### Similarity metric analysis

SIM metric is used to evaluate the spatial overlap between visual features and fixation distributions at the local-region level. As shown in Table 2, SIM values under the free-viewing task are generally higher than those under goal-directed search across most visual features. Among them, high-luminance features, orientation features, and shape and spatial-location features exhibit relatively higher SIM values, suggesting that these features show stronger spatial correspondence with fixation distributions during free viewing.

**Fig 8:**
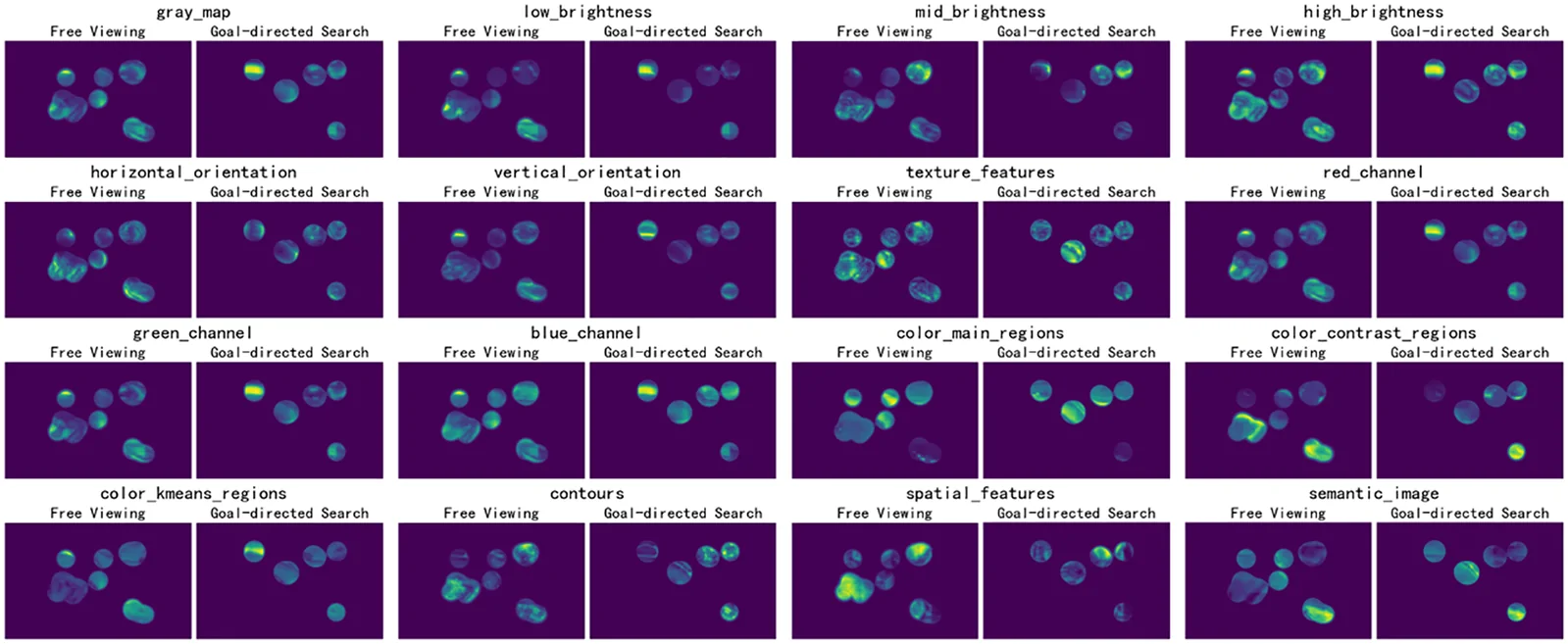
Visualized SIM similarity maps of saliency and fixation distributions for free viewing vs goal-directed search. SIM metric heatmaps illustrating spatial overlap between low-, mid-, and high-level visual saliency representations and human fixation distributions. Paired subplots separately display results of free viewing and goal-directed search tasks for one individual participant and a single test image.

Fig 8 illustrates an example of the spatial distribution of SIM values for different features under the free-viewing and target-search tasks, computed from the fixation heatmap of a single participant on an exemplary image. All visualization results share the identical sample stimulus shown in Fig 3. The image uses the viridis color map, where bright yellow indicates regions with higher SIM values, signifying strong spatial overlap between visual feature distributions and human fixation distributions, while dark purple represents regions with SIM values close to zero or relatively low that indicate little spatial matching. Overall, the spatial distribution patterns of SIM values do not show substantial differences across different visual features.

### Normalized scan path saliency analysis

The NSS metric describes the average response strength of a feature saliency map at human fixation points. Higher NSS values mean a stronger positive correlation between the feature map and the fixation distribution. An NSS value near zero shows little to no correlation, and a negative NSS means the feature map is inversely related to fixation distribution, with inconsistent spatial patterns and poor matching.

As shown in Table 2, orientation features and shape–spatial position features have relatively high NSS values, indicating a strong positive correlation with fixation distributions. Single-color channels and color-segmented regions show a weak negative correlation with fixation distributions. Contrast-color regions have a higher positive correlation with fixation distributions than main-tone regions.

**Fig 9:**
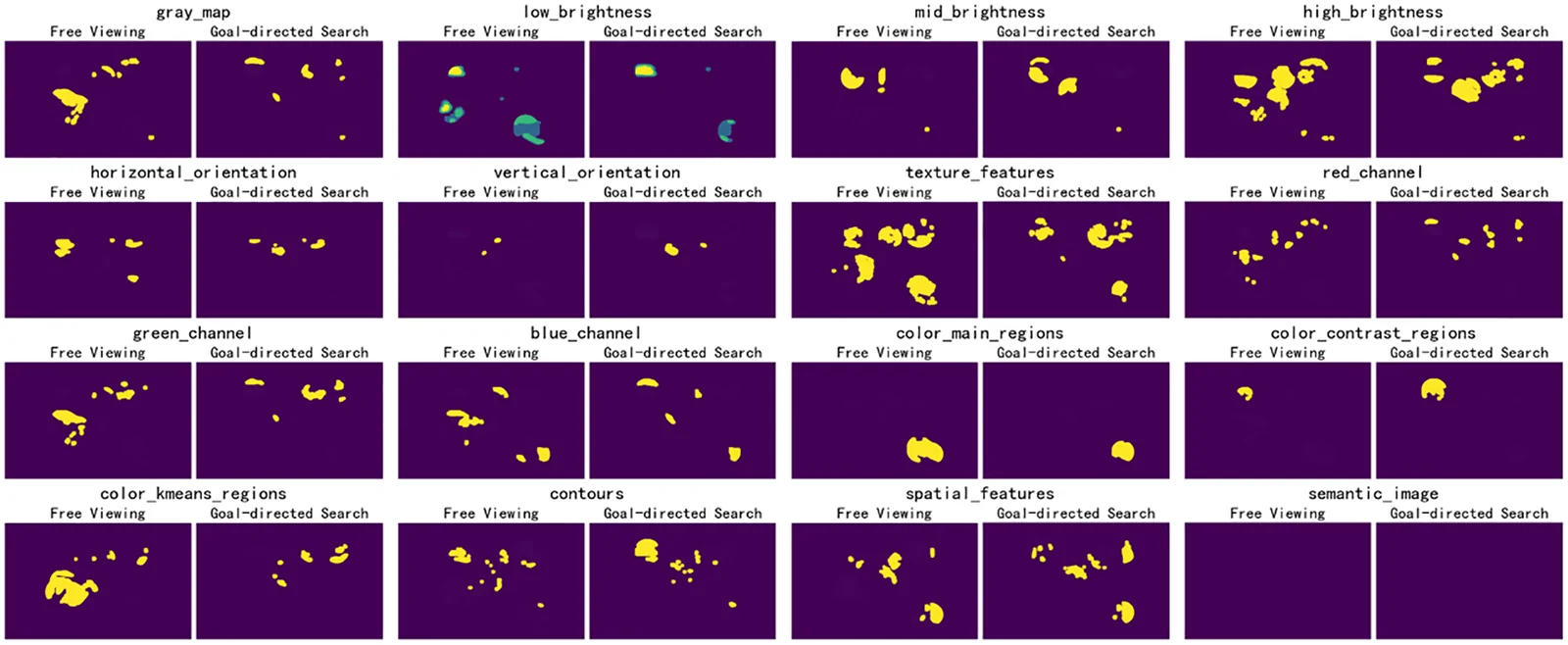
Visualized NSS score maps of saliency and fixation distributions for free viewing vs goal-directed search. NSS heatmaps showing how well multi-level image saliency predicts human fixation locations. Each visual feature is split into two panels to directly compare gaze guidance strength under free viewing and goal-directed search in one representative trial.

Fig 9 illustrates an example of the spatial distribution of NSS values for different features under the free-viewing and target-search tasks, computed from the fixation heatmap of a single participant on an exemplary image. All visualization results share the identical sample stimulus shown in Fig 3. The image uses the viridis color map, where bright yellow represents regions with higher positive NSS values, which reflect strong spatial alignment between visual feature maps and human fixation distributions; green indicates NSS values close to zero, and dark purple represents negative NSS values that indicate inconsistent spatial matching. Compared with free-viewing, the target-search condition tends to produce more localized high-NSS regions, suggesting a more task-driven fixation pattern.

### Information gain analysis

Since human fixations naturally show a center bias, information gain (IG) measures how much feature-based saliency distributions guide fixations beyond the Gaussian center-bias baseline. Higher IG values mean the feature provides stronger guidance for human gaze, going beyond the inherent tendency of observers to fixate near the image center.

Table 2 shows that shape and spatial-location features, together with orientation-related features, have relatively high IG values. These features yield slightly higher IG values in the target-search (SE) condition than in the free-viewing (FV) condition, which may relate to their importance for object recognition during search.

**Fig 10:**
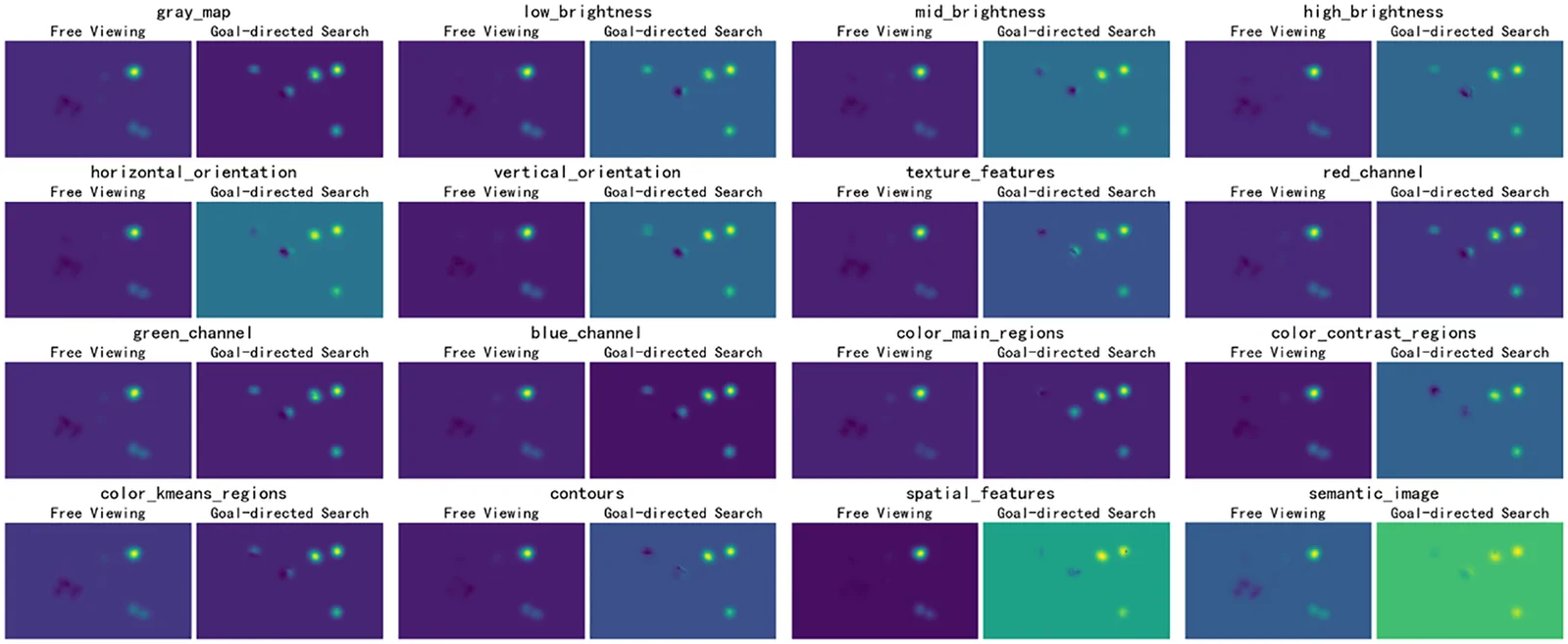
Visualized InfoGain maps of saliency and fixation distributions for free viewing vs goal-directed search. Information gain heatmaps measure feature-specific gaze guidance after removing natural fixation center bias via a Gaussian baseline. Paired subplots compare IG spatial distributions between free viewing and goal-directed search across all visual features for one participant and stimulus image.

Fig 10 presents an example of the spatial distribution of IG values for different features under the free-viewing and target-search tasks, computed from the fixation heatmap of a single participant on an exemplary image. All visualization results share the identical sample stimulus shown in Fig 3. The image uses the viridis color map, where bright yellow represents regions with higher IG values, which reflect areas where visual features provide more effective predictive cues for human fixations, while dark purple indicates values close to zero. Overall, the goal-directed search condition has slightly more high-IG regions than the free-viewing condition. In particular, spatial-location and semantic features exhibit higher IG values across the image during goal-directed search, suggesting stronger guidance in directing visual attention.

### Eye movement trajectory analysis results

**Fig 11:**
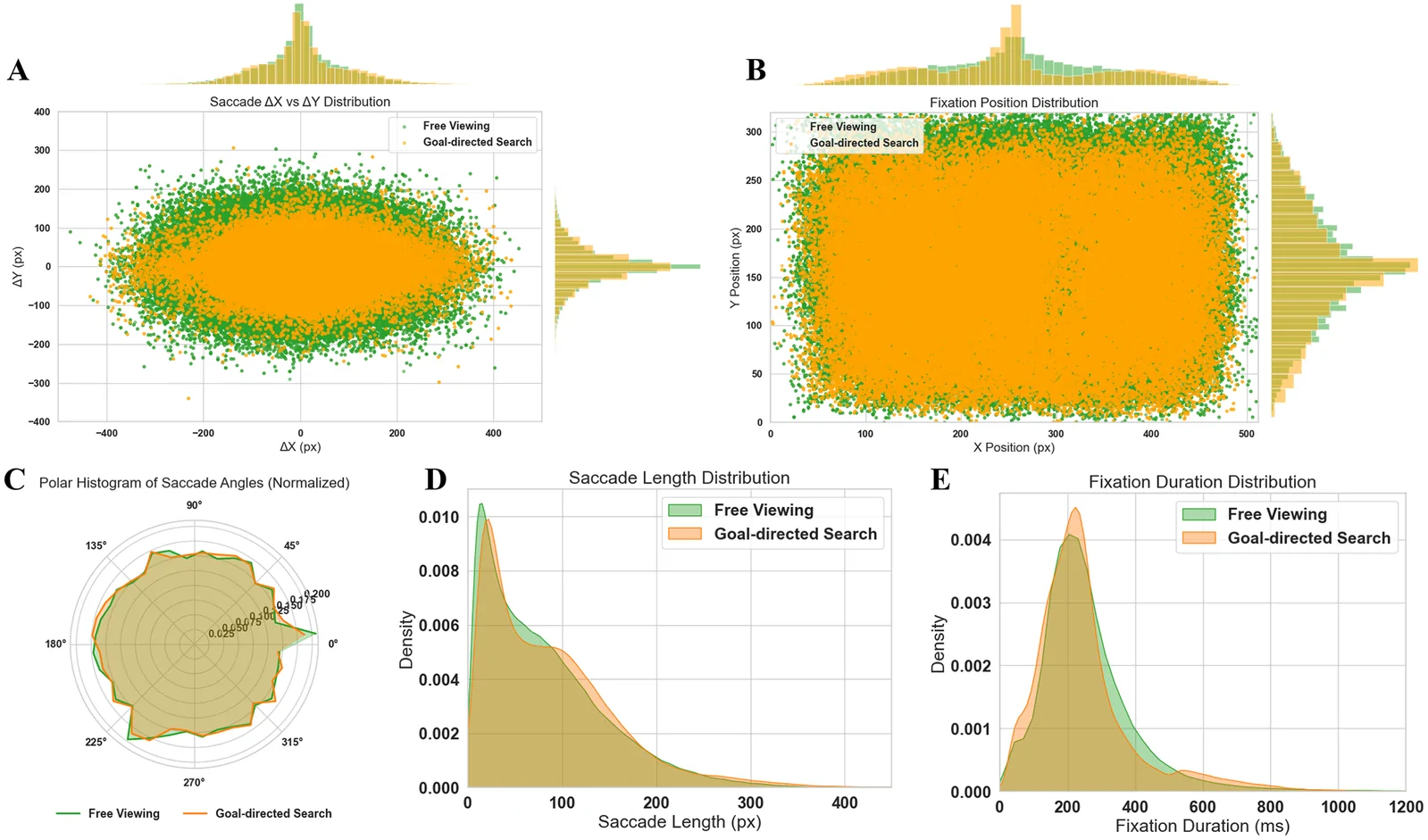
Quantitative distributions of scanpath metrics for free viewing and goal-directed search tasks. Multi-panel visualization of eye movement scanpath features contrasting free viewing (green) and goal-directed search (orange). (A) Scatter plot of horizontal and vertical saccade displacements with marginal histograms; (B) Spatial distribution of all fixation coordinates across stimulus frames with X/Y marginal histograms; (C) Normalized polar histogram showing angular distribution of saccade directions; (D) Probability density function of saccade amplitude in pixels; (E) Probability density function of fixation duration in milliseconds.

The trajectory characteristics under the two viewing tasks are illustrated in Fig 11. All visualized eye-movement statistics are derived from an initial total of 43,978 raw eye-movement trajectories recorded under the free-viewing task and another 43,978 raw trajectories collected during the goal-directed search task across all stimuli and participants. After excluding 500 invalid entries containing NaN values per group, 43,478 valid trajectories remained for statistical analysis in each viewing condition. The original experimental stimuli were sized 1024 × 640 pixels; to reduce computational overhead, all images were downsampled to a resolution of 512 × 320 pixels, and corresponding eye-tracking coordinates were rescaled to match this reduced frame. Panel (A) shows the joint density distribution of horizontal (ΔX) and vertical (ΔY) pixel offsets of individual saccades; (B) displays the spatial distribution of all fixation coordinates mapped onto the 512 × 320 stimulus frame, with marginal histograms on the top and right revealing a clear central bias in human gaze allocation; (C) presents the probability density distribution of saccade angles (°), saccade angles and lengths are calculated from horizontal and vertical saccade offsets: angles start at 0° pointing right; (D) shows the probability density distribution of saccade amplitudes (px) derived from the Euclidean length formula; and (E) presents the probability density distribution of fixation durations (ms). Green traces represent the free-viewing condition, while yellow traces represent the goal-directed search condition. Under the free-viewing task, fixation locations appear slightly more widely distributed across the image, saccade distances along the y-axis tend to be larger, saccade directions are somewhat more concentrated, and fixation durations are generally shorter.

We further conducted one-sample t-tests, 10,000-iteration sign-flipping permutation tests, and calculated standardized Cohen’s d effect sizes based on the 43,478 valid eye-movement trajectories from the free-viewing condition and another 43,478 valid trajectories from the goal-directed search condition. All statistical analyses were carried out on inter-task metric differences computed as goal-directed search values minus free-viewing values, and all test outcomes are summarized in Table 3. We conducted both statistical procedures independently for each eye movement metric. For permutation tests, the null hypothesis assumes no consistent systematic difference in eye movement metrics between the two viewing tasks. All six metrics revealed statistically significant deviations from a mean difference of zero.

**Table 3:** Statistical test results for eye-movement differences.

Eye movement metric	Test type	Statistic	P-Value	Cohen’s d	Permutation mean difference	Permutation P-value
Horizontal saccade offset	t-test	212.3013	$<1\times 10^{-16}$	1.018	–	–
	permutation	–	–	–	0.0785	$<1\times 10^{-16}$
Vertical saccade offset	t-test	221.9555	$<1\times 10^{-16}$	1.064	–	–
	permutation	–	–	–	0.0751	$<1\times 10^{-16}$
Saccade direction	t-test	593.1282	$<1\times 10^{-16}$	2.845	–	–
	permutation	–	–	–	0.4250	$<1\times 10^{-16}$
Saccade amplitude	t-test	391.9023	$<1\times 10^{-16}$	1.880	–	–
	permutation	–	–	–	0.0814	$<1\times 10^{-16}$
Fixation position	t-test	391.9023	$<1\times 10^{-16}$	2.544	–	–
	permutation	–	–	–	0.1845	$<1\times 10^{-16}$
Fixation duration	t-test	493.7605	$<1\times 10^{-16}$	2.368	–	–
	permutation	–	–	–	0.3787	$<1\times 10^{-16}$

All tests are performed on metric differences calculated as goal-directed search minus free viewing. We conducted one-sample t-tests and 10,000 sign-flipping permutation tests for each eye movement metric. Horizontal/vertical saccade offset denote the horizontal and vertical displacement components of saccades, while saccade direction, saccade amplitude, fixation position and fixation duration adopt the exact scanpath metric terminology defined in Section 2.2 of the Methods. The null hypothesis of permutation tests assumes no consistent systematic difference in eye movement metrics between the two viewing tasks. Cohen’s d is the standardized effect size only reported for t-test outcomes; an en dash “–” indicates not applicable for that statistical index.

Our data contains 43,478 paired scanpath entries from the two tasks, providing abundant repeated eye-tracking observations. Large sample sizes inherently inflate statistical significance, and all t-test p-values reached the lower bound of $<1\times 10^{-16}$. For this reason, we prioritize interpreting Cohen’s d effect sizes rather than p-values to quantify the practical magnitude of task-driven eye movement differences. We followed standard classification thresholds widely adopted in behavioral vision research to contextualize effect size strength: |d|<0.2 (trivial), 0.2≤|d|<0.5 (small), 0.5≤|d|<1.2 (moderate), 1.2≤|d|<2.0 (large), and |d|>2.0 (very large).

Three metrics showed very large effects (|d|>2.0): saccade direction (d=2.845), fixation position (d=2.544), and fixation duration (d=2.368). Saccade amplitude yielded a large effect (d=1.880), while horizontal and vertical saccade offsets had moderate-to-large effects (d=1.018 and d=1.064, respectively). These outcomes demonstrate that task context produces strong, practically meaningful changes to scanpath patterns.

**Fig 12:**
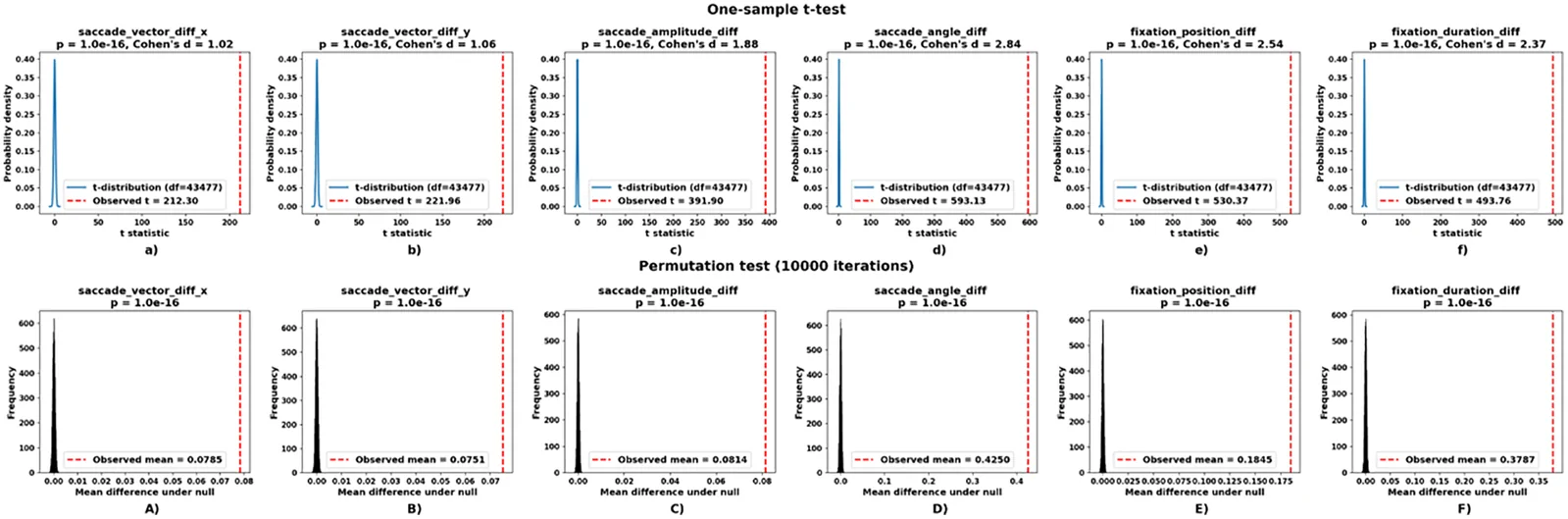
Statistical comparisons of scanpath metrics between free viewing and goal-directed search tasks. Two rows of subplots show statistical test results for six eye movement features: horizontal saccade vector difference (a/A), vertical saccade vector difference (b/B), saccade amplitude difference (c/C), saccade angle difference (d/D), fixation spatial position difference (e/E), and fixation duration difference (f/F). Top row (a–f): One-sample t-test probability density curves, with blue lines denoting the theoretical t-distribution and red dashed lines marking the observed t-statistic; effect size (Cohen’s d) and p-value are labeled above each panel. Bottom row (A–F): Null-distribution histograms from 10,000-iteration permutation tests, where red dashed lines indicate the empirical mean inter-task difference for each scanpath metric, with p-values annotated above each subplot. All features yield highly significant inter-task differences ($<1\times 10^{-16}$).

Fig 12 graphically visualizes the one-sample t-tests and 10,000-iteration sign-flipping permutation test results summarized in Table 3, based on the identical eye-movement dataset presented in Fig 11. The null hypothesis assumes there exists no consistent systematic difference in eye-movement scanpath metrics between the free-viewing and goal-directed search tasks; any observed deviation of metric means is attributed to random noise. Lower-case panels (a–f) show one-sample t-tests for horizontal and vertical saccadic displacements, saccade amplitude (px), saccade angle (°), fixation position (px), and fixation duration (ms), with blue curves representing the theoretical t-distribution under the null hypothesis and red dashed lines indicating the observed t values; exact p-values and Cohen’s d effect sizes are annotated within each subplot. Upper-case panels (A–F) show the results of permutation tests (10,000 iterations), with black curves representing the null distribution of mean differences generated by 10,000 random sign-flipping permutations, and red dashed lines indicating the observed mean differences. Quantitatively, all six trajectory metrics produce extremely small p-values and large Cohen’s d effect sizes, rejecting the null hypothesis and confirming robust systematic disparities in scanpath behaviors between free viewing and goal-directed target search rather than relying on visual qualitative judgment alone.

### Model classification results

As shown in Table 4, among traditional models, MLP performed best across all evaluation metrics, followed by Random Forest and AdaBoost. Naive Bayes showed the weakest performance on all indicators. These differences may come from the models’ different abilities to capture nonlinear and high-dimensional patterns in eye movement features. MLP can model complex relationships between features, while ensemble methods like Random Forest and AdaBoost improve robustness and prediction performance by combining multiple base learners. In contrast, Naive Bayes relies on the assumption of feature independence, which rarely holds for correlated gaze features, and this may account for its relatively poor performance.

**Table 4:** Classification metrics across different models.

Model	Accuracy	Precision	Recall	F1 Score
SVM	0.8743	0.8771	0.8743	0.8743
Decision Tree	0.9229	0.9247	0.9229	0.9229
Random Forest	0.9524	0.9293	0.9234	0.9234
MLP	0.9548	0.9554	0.9548	0.9548
AdaBoost	0.9444	0.9459	0.9444	0.9445
Logistic Regression	0.8764	0.8773	0.8764	0.8762
kNN	0.9379	0.9393	0.9379	0.9379
Naive Bayes	0.7649	0.7978	0.7649	0.7559
**CNN-TransGaze**	**0.9727**	**1.0000**	**0.9700**	**0.9900**

**Fig 13:**
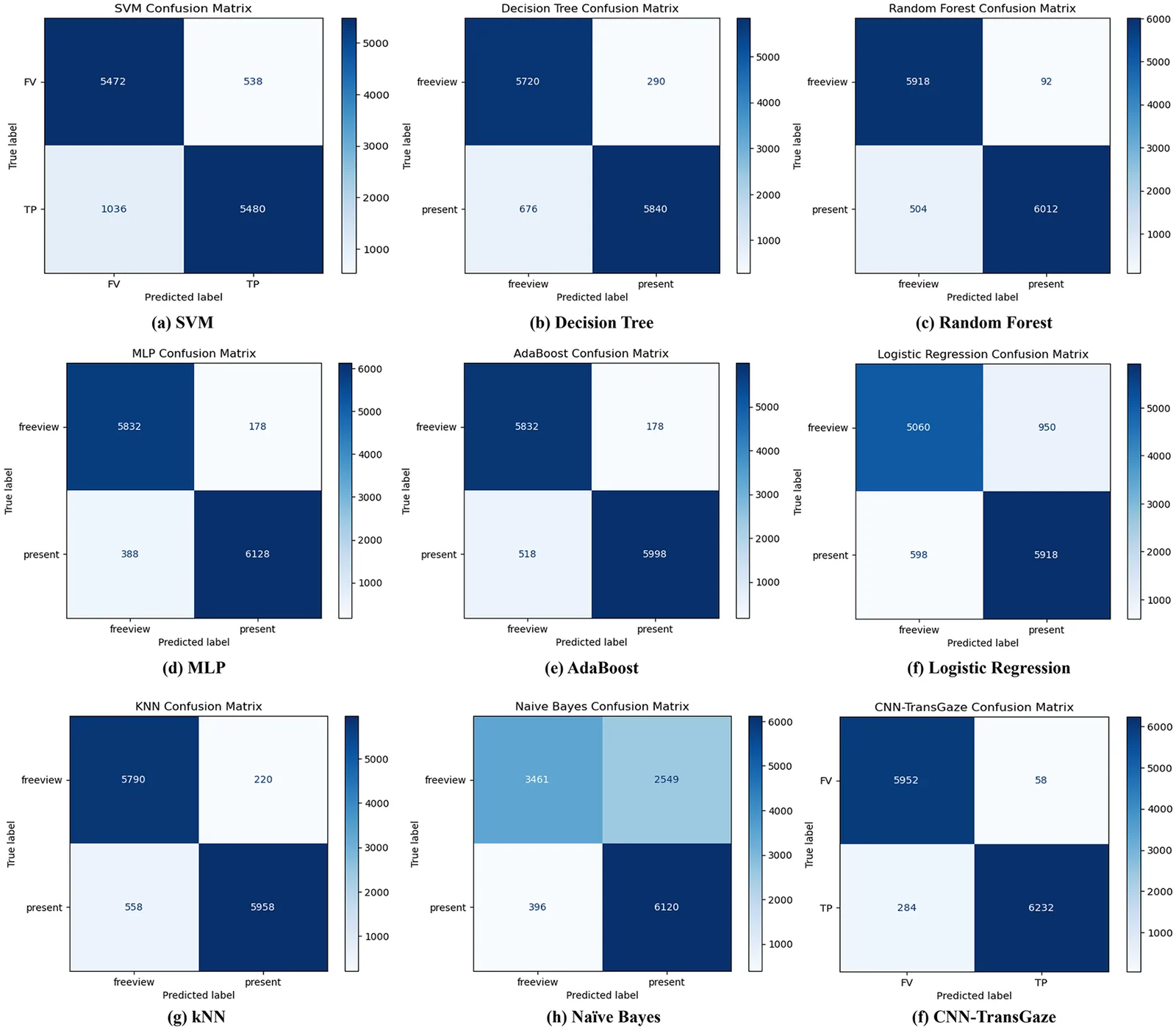
Confusion matrices for all baseline classifiers and the proposed CNN–TransGaze model. Nine confusion matrices illustrating binary classification performance to distinguish free viewing (freeview) and goal-directed search (present) gaze patterns: (a) SVM, (b) Decision Tree, (c) Random Forest, (d) MLP, (e) AdaBoost, (f) Logistic Regression, (g) kNN, (h) Naïve Bayes, and (i) our CNN–TransGaze hybrid framework. The proposed CNN–TransGaze model achieves the lowest misclassification counts compared to all traditional machine learning baselines.

Among all indicators, the deep learning model CNN-TransGaze performs best, with an accuracy of 0.9727, recall of 0.9700, F1-score of 0.9900, and precision of 1.0000. This result aligns with MLP being the best among traditional methods, showing that deep learning can effectively extract high-dimensional features from gaze data and yields better recognition performance than handcrafted statistical features. Fig 13 presents the confusion matrices of all evaluated models.

**Fig 14:**
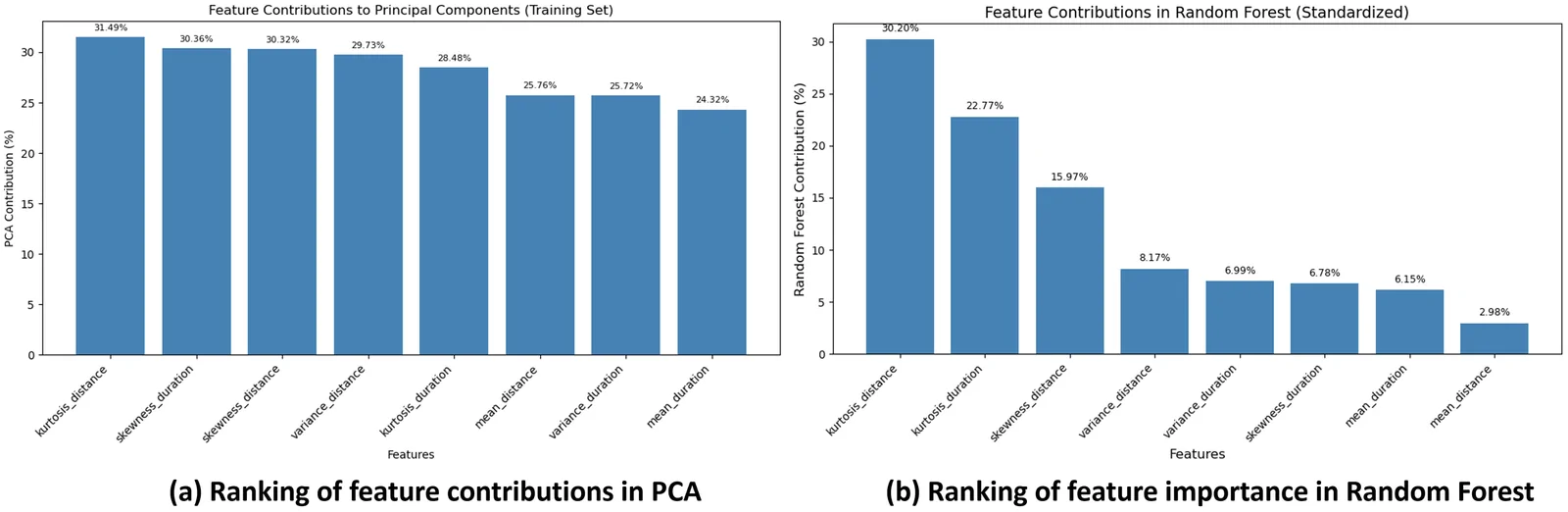
Quantitative ranking of eye movement distributional features via PCA contribution and random forest feature importance. Bar charts comparing feature weight rankings derived from two analytical approaches on gaze distribution metrics. (a) Relative feature contribution percentages to principal components calculated on the training dataset; (b) Standardized feature importance scores (%) extracted from the trained Random Forest classifier. Kurtosis of saccade distance exhibits the highest contribution and predictive weight under both methods.

Principal Component Analysis (PCA) was conducted on the eight eye movement features, with results shown in Fig 14(a). The PCA results indicate that saccade amplitude kurtosis contributes most strongly to the principal components (31.49%), followed by fixation duration skewness (30.36%) and saccade amplitude skewness (30.32%). Feature importance analysis from the Random Forest model (Fig 14(b)) also indicates that saccade amplitude kurtosis is the most important predictor, representing 30.20% of total importance. It is followed by fixation duration kurtosis (22.77%) and saccade amplitude skewness (15.97%).

## Discussion

This study consists of three sequential analytical modules that progress from intrinsic feature-driven mechanisms to overt behavioral manifestations and ultimately automated pattern recognition. First, we quantify the competitive gaze-attraction effects of multi-layer visual features under free viewing (dominated by bottom-up attention) and goal-directed search (dominated by top-down attention), revealing the fundamental feature-based drivers of attentional allocation. Building on these mechanistic findings, we next characterize overt behavioral disparities by extracting multi-dimensional scanpath metrics and applying statistical tests to systematically delineate surface-level oculomotor variations induced by distinct task requirements. Finally, drawing on the verified inter-group behavioral differences, we compare the discriminative power of conventional handcrafted eye-movement statistics against latent representations learned via deep learning. Our results confirm that temporal deep models capture subtle spatiotemporal gaze signatures inaccessible to manual feature engineering and yield superior performance in distinguishing task-specific eye movement patterns.

### Hierarchical contributions of visual features to gaze guidance

In Section 3.1, mid-level structural features such as shape and spatial distribution agreed well with human fixation patterns across all measures, suggesting that structural cues play a key role in guiding gaze behavior [20–22]. This may be because shape and spatial position facilitate rapid extraction of scene organization, enabling observers to efficiently identify informative regions [23].In addition, orientation-related features also showed notable associations with fixations [24,25], likely because horizontal and vertical orientation cues rapidly define the global layout of a scene [26]. The influence of contour and spatial location on visual attention can be understood within a hierarchical processing and competitive selection framework. In early visual cortex (V1), neurons encode local edge features within a retinotopic organization, forming an initial spatially structured representation of visual input [27]. These edge signals are then integrated into continuous contours in V2 and higher visual areas via horizontal and feedback connections, and are further assigned object-specific ownership through the border ownership mechanism, transforming low-level features into object-boundary representations [27,28]. Behavioral evidence further shows that spatial cues significantly enhance processing efficiency at corresponding locations even when stimulus features remain unchanged, indicating that attention is initially allocated according to spatial coordinates [29]. Collectively, these findings suggest that visual attention operates via a spatial priority map, where spatial location provides the coordinate framework and contour information imposes object-level constraints that jointly guide selection.

In contrast, other features such as color, luminance, and texture [30], exhibited relatively weaker associations with fixation locations. This may be because these features mainly represent local visual properties, while observers usually focus more on global structural information than local details when looking at natural scenes [31]. Semantic features, in contrast, exhibited task-dependent effects, with their influence on fixation patterns varying across viewing conditions.

### Task context modulates the guidance of visual features

The results indicate that task context significantly modulates the influence of visual features on fixation behavior, particularly for semantic features. During free-viewing tasks, semantic features showed only limited association with fixation locations, whereas in goal-directed search tasks, their influence on gaze patterns was markedly stronger. This may be because goal-directed task demands enhance the use of semantic information, thereby increasing its role in guiding gaze behavior [32]. Neurophysiological and behavioral evidence indicates that the human visual system can rapidly extract the global semantic gist of a scene and use this information to constrain subsequent attentional allocation, such that objects consistent with scene semantics or behaviorally relevant entities are more likely to attract fixation [33,34]. The key difference between free-viewing and goal-directed search lies in the underlying attentional control regime: the former relies on a weakly constrained integration of semantic information and visual saliency, whereas the latter is governed by a strongly constrained, template-driven selection mechanism. In goal-directed search, target representations are actively maintained in visual working memory and interact with long-term semantic memory to form a target template, which enhances the processing gain of stimuli matching task-relevant semantic features while suppressing irrelevant information, thereby significantly improving search efficiency [35].

In contrast, for other visual features, associations with fixations were generally stronger during free-viewing than during search tasks. Observers in the free-viewing condition relied more on physical salience and structural features [21], exhibiting an exploratory viewing pattern. They tended to sample information broadly across the scene rather than focusing on specific regions.

These task-dependent differences in feature guidance may be related to differences in attentional control across viewing conditions. Specifically, free-viewing is largely driven by bottom-up attention, such that observers are more readily attracted by bottom-up physical salience, leading to an exploratory gaze pattern. Conversely, goal-directed search is dominated by top-down attentional control, which prioritizes task-relevant information over generic physical stimulus properties.

### Task context modulates eye movement patterns

In addition to differences in visual feature guidance, eye movement patterns also differ significantly across task conditions. During free viewing, eye movement behavior is more driven by intrinsic visual exploration tendencies [36,37], with larger saccade amplitudes [23,38], shorter fixation durations that are also influenced by peripheral information [39], and more spatially dispersed distributions [21,23]. Attention allocation relies more on immediate perceptual input and other visual features [23].

In contrast, during goal-directed tasks, observers tend to concentrate their fixations on regions that are likely to contain target-relevant information [21], characterized by smaller and more consistent saccades [40,41], as well as more spatially concentrated and longer fixations [42,43]. This may be because task goals continuously modulate fixation distributions [44,45], constrain attentional resources within a limited spatial range of visual exploration [46], and optimize gaze trajectories to maximize the acquisition of task-relevant information [45,47–49].

### Latent high-dimensional feature extraction via deep learning

Higher-order distributional features (e.g., kurtosis and skewness) show greater effectiveness in distinguishing eye movement patterns under different task conditions. This may be because the relevant differences in eye movement behavior are more strongly reflected in systematic changes in data distribution patterns, rather than being determined solely by average measures (e.g., mean fixation duration, mean saccade amplitude). For example, during goal-directed search, eye movements typically exhibit stronger spatial concentration (e.g., fixations clustered in task-relevant regions) and higher distributional asymmetry (e.g., a higher proportion of longer fixations) [50,51], whereas under free viewing conditions, eye movements tend to be more dispersed and less structured [52,53].

Compared with methods based on manually extracted statistical features, deep learning methods can directly learn latent high-dimensional representations from raw eye movement sequences [54], thereby capturing more complex spatiotemporal patterns. Previous studies have shown that deep learning has been widely applied to eye movement trajectory prediction [55,56], scene saliency prediction [57], and spatiotemporal feature extraction from eye-tracking data [58], further demonstrating its advantages in handling complex eye movement data. This ability to extract latent features is particularly effective in distinguishing different eye movement behavior patterns. For example, in differentiating eye-movement patterns between healthy individuals and patients with neurodegenerative or attentional disorders, healthy participants performing goal-directed visual search typically rely on top-down task goals to suppress irrelevant low-level saliency cues and preferentially fixate mid-level shape and semantic features relevant to targets. In contrast, individuals with attentional deficits show impaired top-down control and increased susceptibility to bottom-up saliency, resulting in frequent fixation shifts toward irrelevant but visually conspicuous stimuli and reduced task-relevant exploration efficiency [59]. Our study establishes a comprehensive normative baseline of hierarchical feature fixation preferences and standardized temporal eye-movement representations derived from healthy observers under both free-viewing and goal-directed search. The learned representations and pre-trained weights can further provide a transferable foundation for downstream applications in cognitive decline-related disease screening, offering a data-driven normative reference for early cognitive impairment detection.

## Conclusion

This study investigates how visual features guide gaze behavior and how eye movement patterns differ across task conditions (free viewing vs. goal-directed search) and further develops a model to classify eye movement patterns. The results show that features such as shape, spatial location, and orientation exhibit significant attraction to fixation under both task conditions. Under free viewing conditions, these visual features show stronger guidance in gaze allocation, whereas under goal-directed search conditions, semantic information plays a more prominent role in guiding fixations, which may be related to its involvement in target localization. In addition, significant differences in eye movement patterns are observed across the two task conditions. Higher-order statistical features demonstrate greater sensitivity in distinguishing these differences, suggesting that task-related variations are primarily reflected in the distributional structure of eye movement behavior rather than in average measures alone. Furthermore, deep learning models can learn latent high-dimensional representations from raw eye movement data, which enables more effective discrimination of eye movement patterns across task conditions. Considering that patients with neurodegenerative diseases exhibit significantly distinct oculomotor patterns compared with healthy controls during both task paradigms, the model parameters learned from healthy populations in this work can serve as a reliable self-supervised pre-trained backbone for future clinical screening. Furthermore, the proposed quantitative frameworks for evaluating feature gaze guidance and analyzing scanpath differences provide effective analytical tools to objectively measure behavioral discrepancies between healthy individuals and clinical patients in subsequent neurological studies.
